# Ivabradine in Congestive Heart Failure: Patient Selection and Perspectives

**DOI:** 10.7759/cureus.4448

**Published:** 2019-04-13

**Authors:** Yasar Sattar, Elham Neisani Samani, FNU Zafrullah, Sharaad Latchana, Nirav B Patel

**Affiliations:** 1 Internal Medicine, Icahn School of Medicine at Mount Sinai, New York, USA; 2 Internal Medicine, Yale University, New Haven, USA; 3 Internal Medicine, Steward Carney Hospital, Tufts University School of Medicine, Boston, USA; 4 Internal Medicine, American University of Integrative Sciences, Tucker, BRB; 5 Internal Medicine, Lasante Health, Jersey City, USA

**Keywords:** heart failure, heart failure with reduced ejection fraction (hfref), ivabradine, nyha functional class, mortality, heart failure decompensation, heart rate, rehospitalization

## Abstract

Heart failure (HF) is the fourth-most frequent cause of death and remains a challenge for public health. Therapy goals for HF with reduced ejection fraction (HFrEF) are the improvement in the quality of life, prolonged survival, a reduction of signs and symptoms, and the prevention of hospitalization. Angiotensin-converting enzyme inhibitors, beta‐blockers, and mineralocorticoid receptor antagonists are the treatments of choice for HFrEF. Although ivabradine is not available in all countries, it is likely a new promising approach to improve outcomes in patients with HFrEF, either alone or with beta-blockers. Here, we review the current knowledge about ivabradine in HFrEF and assess its effect on outcomes in HF.

## Introduction and background

Congestive heart failure (CHF) remains a debilitating chronic condition with a notable economic burden. In the United States, more than 6.5 million people have CHF, and around 650,000 new patients are diagnosed every year. It is significantly prevalent in the elderly population, and CHF is a leading cause of impaired daily life, hospitalization, morbidity, and mortality [1].

Heart failure (HF) with reduced ejection fraction (EF; <40%) (HFrEF) occurs in approximately 50% or more of patients with CHF. Over the past decade, the management of and therapeutic options for HFrEF have improved; however, many patients are still faced with critical complications [2]. Currently, patients with HFrEF benefit from heart failure cocktail medications like beta-blockers, angiotensin-converting enzyme (ACE) inhibitors, angiotensin receptor type 1 antagonists, aldosterone receptor antagonists, diuretics, and digoxin. In a randomized, double-blind, placebo-controlled, parallel group study, more than 6,500 patients with HFrEF (EF < 35%) in sinus rhythm and with a heart rate greater than 70 beats per minute (bpm) were randomized to ivabradine or placebo. During 23 months of monitoring, HF hospitalizations were decreased by 26% in patients treated with ivabradine [3-4]. As a result of this study, ivabradine was approved with an indication for use in combination with a beta-blocker in patients with HFrEF with persistently symptomatic inappropriate sinus tachycardia. It is not clear whether ivabradine can affect cardiovascular mortality in this group of patients. The long-term efficacy and safety of ivabradine are not yet known. Major trials have demonstrated an increased risk of atrial fibrillation and phosphenes, leading to the stoppage of the medication [4-5].

In this review, we summarize evidence connecting the possible risks and benefits of ivabradine in patients with HFrEF.

## Review

Methods

To search the literature for information on the efficacy, effectiveness, dosage, and safety of ivabradine in patients with HFrEF, we reviewed related publications in Medline, Cochrane, Ovid, and Embase databases from January 1980 to January 2019. The following broad research terms, including medical subject headings (MeSH), were used: Heart Failure, Heart Failure with Reduced Ejection Fraction (HFrEF), Ivabradine, NYHA Functional Class, Mortality, Morbidity, Heart Failure Decompensation, and Heart Rate, Rehospitalization. Two investigators performed the literature research. All randomized and non-randomized clinical trials discussed the role of ivabradine and its efficacy in HFrEF. We excluded case reports and studies that did not evaluate the outcomes. The references listed in the included articles were checked to identify other relevant papers for inclusion.

Heart failure

The American College of Cardiology Foundation (ACCF) / American Heart Association (AHA) guidelines define HF as a complex clinical syndrome that results from structural or functional impairment of ventricular filling or ejection of blood [6]. HF is a major public health issue, with a prevalence of over 25 million cases worldwide. Hospitalization due to HF is rising in the United States, particularly in elderly populations [7]. Despite a notable improvement in consequences with medical treatment, rehospitalization remains high, with more than 50% of patients re-hospitalized within six months of discharge [5].

Current guidelines advocate the classification of HF based on left ventricular (LV) ejection fraction (EF). HF with normal LVEF (≥ 50%) is defined as HF with preserved ejection fraction (HFpEF). HF with decreased LVEF (< 35% to 40%) is defined as HFrEF. HF patients with LVEF in the range of 40% to 49% are defined as having HF with mid-range ejection fraction [8-9]. HF is also classified into four types based on symptomatic presentations by New York Heart Association (NYHA), as shown in Table 1.

**Table 1:** New York Heart Association (NYHA) functional classification Abbreviation: HF, heart failure.

Class	Functional Capacity
I	No limitation of physical activity. Ordinary physical activity does not cause fatigue, palpitation, or dyspnea
II	Slight limitation of physical activity. Comfortable at rest. Ordinary physical activity results in fatigue, palpitation, or dyspnea
III	Marked limitation of physical activity. Comfortable at rest. Less than ordinary physical activity causes fatigue, palpitation, or dyspnea
IV	Unable to perform any physical activities without discomfort. Symptoms of HF at rest. If physical activity is undertaken, discomfort increases

Early evaluation of HF should assess natriuretic peptides and echocardiography. Echocardiography provides more information including chamber size, a systolic and diastolic function of ventricles, wall thickness, and valve abnormalities which help to set up a management plan [7]. Novel biomarkers and genetic testing still have room for improvement [3]. Before the 1980s, a patient with HF was treated with fluid restriction, digitals, and diuretics. A large randomized controlled trial of vasodilator treatment in HF published in 1986 found that vasodilator treatment improved mortality [10]. Subsequent studies showed that vasodilator treatment had no benefit for the long-term survival of patients with HF. Then, beta-blockers and ACE inhibitors were identified as able to improve LV function and declined hospitalization rate and mortality [6]. From the 2000s, studies of patients with HFrEF revealed that ivabradine seemed to be more beneficial in patients under beta-blocker therapy with a resting heart rate > 70 bpm [11]. Pharmacologic treatment of HF is focused on controlling symptoms and reducing risk factors and comorbidities. Before the approval of ivabradine, beta-blockers and digoxin were prescribed to reduce heart rate and improve rhythm [12].

Ivabradine

Ivabradine has been used for angina pectoris but was recently approved for the treatment of HFrEF (NYHA category II-IV) patients with a heart rate of ≥ 70 bpm in the United States. It was recommended for use in the treatment of patients with HFrEF (EF of approximately 35% to 40%), in persistent sinus tachycardia who are taking beta-blockers at the maximum dose, ACE inhibitors (or angiotensin II receptor blockers) and magnetic resonance angiography. Indeed, ivabradine is preferred for symptomatic HFrEF in patients who are unable to tolerate or have contraindications to beta-blockers.

Ivabradine is a water-soluble agent with a bioavailability of 40% and intestinal absorption. It is metabolized in the liver and intestine by cytochrome P450 and has a half-life of 11 hours. Ivabradine use is contraindicated in those with severe hepatic impairment. Dose adjustment is not required for renal failure where the creatinine clearance is between 15 to 60 mL/minute [2, 8].

**Figure 1:**
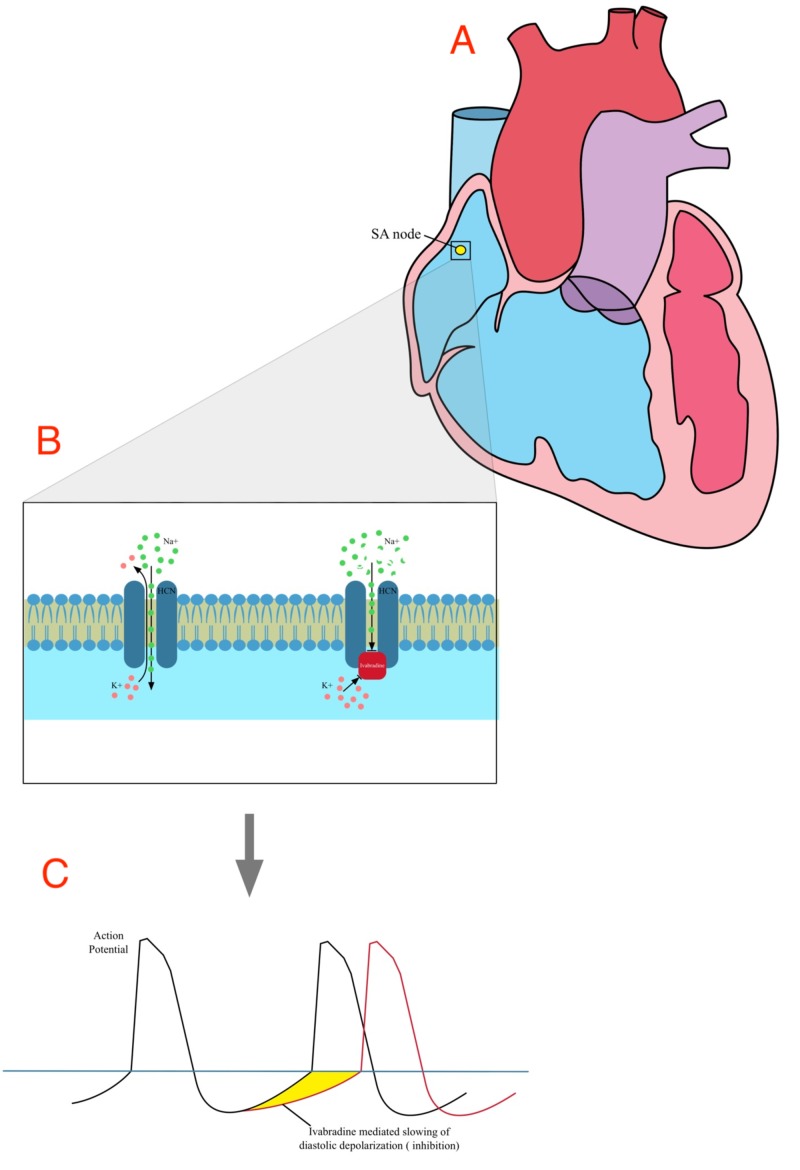
Mechanism of action of ivabradine. A. The sinoatrial node (SAN) is the primary place of action on cardiac tissue. B. Ivabradine blocks the intracellular aspect of the hyperpolarization-activated cyclic nucleotide-gated transmembrane channel which is responsible, in the open state, for the transport of sodium and potassium ions across the cell membrane. This leads to inhibition of the funny current (Iƒ) channel, which is specifically activated at hyperpolarized membrane potentials. C. Ivabradine causes a dose-dependent reduction in heart rate via the mediated slowing of diastolic depolarization (shaded region) and increasing the duration of diastole without altering other phases of the action potential.

Ivabradine binds to a subgroup of hyperpolarization-activated cyclic nucleotide-4 channels including the Iƒ channel. The number of open channels is related to the number of action potentials. Ivabradine is an Iƒ channel inhibitor; inhibition of this channel reduces heart rate but does not affect contractility and ventricular repolarization. The Iƒ channel is a combined sodium/potassium channel, a member of the hyperpolarization-activated, cyclic nucleotide channel family responsible for the spontaneous action potential in the sinoatrial node. The autonomic nervous system regulates the Iƒ channel. Activation of beta-1 receptors in the sinoatrial node increases cyclic adenosine monophosphate (cAMP) levels, which bind to the Iƒ channel [3, 5]. Binding of cAMP shifts the threshold for activation of Iƒ currents to more positive membrane voltages, making it easier for the cell to depolarize quickly. A higher heart rate makes ivabradine more effective [13]. A simplified version of the mechanism of action of ivabradine is shown in Figure 1.

Clinical aspects of ivabradine in HF

Since ivabradine’s approval by the US Food and Drug Administration for use in HF in 2015 and the European Medicines Agency approval in 2012, it remains a third-line therapy in HF. Nevertheless, it is not available in all countries [11]. The number of clinical studies investigating the use of ivabradine is low.

Ivabradine is recommended at a starting dose of 5 mg, twice per day for two weeks. Then, the dose may be adjusted as required; if a patient’s resting heart rate is persistently at or above 70 bpm, then the dose should be increased to 7.5 mg twice daily. If a resting heart rate is less than 60 bpm, the dose is decreased to 2.5 mg twice per day [14]. If the patient’s heart rate remains persistently below 50 bpm despite dose reduction or if the patient develops symptomatic bradycardia, ivabradine should be discontinued [15]. Preferably, ivabradine would be initiated by a cardiologist with access to a multidisciplinary HF team to facilitate dose adjustment [16].

Ivabradine is a costly treatment for HF. In the manufacturer’s base case probabilistic analysis, the incremental cost-utility ratio for ivabradine plus standard of care (SOC) was $7,969 per quality-adjusted-life-year compared with SOC alone [2].

The safety and efficacy of ivabradine were studied in a randomized, double-blinded placebo-controlled clinical trial of 6,505 participants in 2010 (in the Systolic Heart Failure Treatment with the IF Inhibitor Ivabradine Trial; SHIFT). Patients with symptomatic HFrEF who admitted to the hospital for worsening HF within the previous 12 months had a sinus rhythm of ≥ 70 bpm. The starting dose of the study drug, 5 mg, was raised to 7.5 mg twice daily unless the patient’s resting heart rate was 60 bpm or lower. The significant adverse effects noted were mild symptomatic bradycardia, atrial fibrillation, and phosphenes. This study showed that ivabradine significantly reduced readmission for worsening HFrEF, whereas mortality was not affected. In the SHIFT study, only 26% of patients were at an optimal dose of beta-blockers, 56% were at >50% of the target dose of beta-blockers, and 11% were on no beta-blocker [4, 17]. Hypotension was reported as the main reason for the inability to reach the target dose. In a post hoc analysis of the SHIFT study, the effects of ivabradine were compared across five categories of beta-blocker intake (no beta-blocker, <25% target dose, 25% to 50% target dose, 50% to 100% target dose and ≥100% target dose of beta-blockers). There were similar trends in efficacy for ivabradine compared with placebo for the outcomes of readmission and cardiovascular death across the beta-blocker categories; further statistical analysis demonstrated that the treatment effect of ivabradine could explain the magnitude of heart rate reduction rather than the beta-blocker dose [4,10]. Hu et al. evaluated the overall result of ivabradine on 6,558 patients with HFrEF in a randomized, double-blind, placebo-controlled study [16]. This study found the ivabradine improved clinical prognosis and quality of life, as well as reduced heart rate in Chinese patients with HFrEF without reported side effect [16]. Tondi et al. retrospectively analyzed the clinical charts of 308 patients with HFrEF over 68 months [1]. Of 308 HFrEF patients, 220 (71%) were on beta-blocker therapy (heart rate was 67 ± 10 bpm). Of the remaining 88 patients, 10 (3.2%) were on a maximally tolerated beta-blocker and ivabradine; 21 patients (6.8%) had heart rates ≥ 70 bpm and EF ≤35% (despite being on maximally tolerated beta-blocker dose), and were, therefore, eligible for ivabradine treatment. Fifty-seven (18%) patients were not on a beta-blocker due to either intolerance or major contraindications. Among them, 13 (4%) were taking ivabradine alone [1]. Of the 44 (14%) patients, 27 (9%) were revealed to have inadequate heart rate control (74 ± 6 bpm). Of these, only eight (3%) patients were determined to be eligible for ivabradine introduction according to heart rate and EF parameters. Tondi et al. found that, in patients with moderate HFrEF, ivabradine was indicated in around 17% of the patient population [1]. While an analysis of pooled prospective studies determined the clinical efficacy of ivabradine in patients with HFrEF, there is no study that evaluates the placebo effect on symptoms [1, 18].

Ivabradine is the drug of choice in patients with HFrEF with chronic obstructive pulmonary disease and asthma, due to the risk of bronchoconstriction from beta-blockers [19]. Ivabradine is also considered for symptomatic relief of HFpEF. It causes better diastolic filling times and reduces heart rate. This is a finding from small studies; further research is needed to give better insight [20].

A limited number of studies have shown the adverse effects of ivabradine such as visual disturbances, bradycardia, and atrial fibrillation [21]. In randomized clinical studies involving more than 3,500 patients and 800 controls, visual symptoms were reported in 17% (n = 270), while sinus bradycardia of ≤ 55 bpm in 3.2% (n = 53) of all patients on 5 mg to 7.5 mg twice daily. Less than 1% of patients withdrew from therapy because of sinus bradycardia. Ivabradine showed no significant effects on QT interval. Due to the inhibition of the Iƒ channel, ivabradine can cause luminous phenomena (phosphenes). Visual disturbances are transient and led to < 1% of withdrawals (24 of 2545 patients); symptoms resolved during treatment in 77.5% (383 of 491) of patients [11].

Phosphenes were reported in 3% (89) of the cases in the SHIFT trial; these resolved after treatment. In the trial of ivabradine for patients with stable coronary artery disease and left-ventricular systolic dysfunction (i.e., the BEAUTIFUL trial), 0.3% (37) of cases withdrew from the trial due to visual changes. In both the BEAUTIFUL (morBidity-mortality EvAlUaTion of the Iƒinhibitor ivabradine in patients with coronary disease and left ventricULar dysfunction) and SHIFT trials, 13% and 11% subsequently withdrew from treatment, respectively. The incidence of arterial fibrillation was 9% in the ivabradine group compared with 8% in controls in the SHIFT trial. Furthermore, a meta-analysis of 21,171 patients from 11 bodies of research, including both the BEAUTIFUL and SHIFT trials, described an increased relative risk of arterial fibrillation with ivabradine of 15% when compared with controls [19].

Ivabradine has been correlated to improve exercise intolerance, reduced cardiac remodeling, improvement in NYHA HF class, and improved quality of life [22].

There are limited data on the long-term efficacy and safety of ivabradine. Most of the studies were small and did not have enough power. The potential teratogenic effects of ivabradine need to be assessed as many patients are women of reproductive potential. A further multicenter, randomized, placebo-controlled, double-blind study is needed for confirmation.

## Conclusions

HF remains a major health issue throughout the world. Despite this, uncertainty exists about the role of ivabradine on outcomes including morbidity and mortality in patients with HF. Ivabradine continues to be well recognized for improving the quality of life measurements and symptoms. Taken together, ivabradine is an effective first choice in selected patients with HFrEF and heart rates of 70 bpm or higher. It has been associated with an excellent safety profile during its clinical development, post-marketing surveillance, and ongoing clinical trials in adult and elderly patients. However, beta-blockers have proven mortality benefit in chronic HF, and the availability of ivabradine should not prevent the initiation of beta-blockers. Based on the evidence from clinical trials, ivabradine should only be prescribed to patients who are already on the maximum-tolerated beta-blocker dose and with a heart rate that remains 70 bpm or more. Hence, ivabradine may prove useful in patients in sinus rhythm who are intolerant of beta-blockers due to hypotension, fatigue, or reactive airway disease.
